# Bioinformatics analysis of GPS1 expression and biological function in breast cancer

**DOI:** 10.1007/s00432-023-05569-2

**Published:** 2024-01-30

**Authors:** Hong Wei, Zhaocan Niu, Ruixue Ji, Wenwen Jiang, Jiawei Tang, Zhexuan Meng, Xiaoyang Cao, Xinyi Zhang, Xue Liu

**Affiliations:** 1 Jining Medical University, ShanDong, China; 2 Provincial Key Laboratory of Pathology and Pathophysiology, ShanDong, China; 3 Jining Key Laboratory of Pharmacology, Jining Medical University, ShanDong, China

**Keywords:** GPS1, Breast cancer, Prognosis, Immune cell infiltration

## Abstract

G protein pathway suppressor 1 (GPS1) is involved in the development of many diseases including tumors, but its specific regulatory mechanism in breast cancer is not clear. The goal of the present study was to explore the biological effects and underlying mechanism of GPS1 in breast cancer. Public databases were used to analyze GPS1 expression and the relationship with clinicopathological characteristics and prognosis of breast cancer patients, combined with in vitro experiments to analyze the mechanism of action and immune relevance of GPS1 in breast cancer. Data analysis showed that the expression of GPS1 in breast cancer tissues was significantly higher than that in paracancerous tissues (*p* < 0.001), and the receiver operating curve (ROC) revealed a higher diagnostic efficiency (AUC = 0.832). Survival analyses indicated that patients with high GPS1 expression made the prognosis worse in Luminal B, low to intermediate-grade breast cancers. Enrichment analysis showed that GPS1 was involved in the formation of ribonucleoprotein complexes, which dynamically altered the fate of RNA; it could also enhance the responsiveness of the Wnt pathway by interacting with WBP2. In addition, GPS1 expression was closely related to the immune microenvironment. GPS1 knockdown inhibits the proliferation, invasion and migration of MCF7 and MDA-MB-231 cells in vitro. This study suggests that the upregulation of GPS1 is associated with the malignant biological behavior and prognosis of breast cancer and may promote cancer progression. The correlation between GPS1 and the immune microenvironment suggests that it may be a potential target for immunotherapy.

## Introduction

Breast cancer is a heterogeneous group of tumors with complex pathogenesis, and rising morbidity and mortality have made it a major global public health problem(Siegel et al. 2023). Despite advances in early diagnosis and clinical interventions that have significantly reduced breast cancer mortality, breast cancer remains an important threat to women's health(Barzaman et al. 2020). Avoiding over-treatment while establishing a set of personalized and precise treatments based on tumor biomarkers or therapeutic targets is the goal pursued in breast cancer treatment. Therefore, the search for reliable tumor biomarkers or therapeutic targets has become the focus of clinical research in breast cancer.

G protein pathway suppressor 1 (GPS1) is an important component of the COP9 signaling complex (CSN), which is involved in a variety of cell growth and developmental processes. The COP9 signalosome is a conserved protein complex consisting of eight subunits, from CSN1 to CSN8. GPS1 is the largest subunit that constitutes the signalosome, and it is also known as CSN1 (Tsuge et al. 2011; Wei and Deng 2003). GPS1 is involved in the development of many diseases including tumors (Cayli et al. 2012; Bech-Otschir et al. 2001), in vitro experiments revealed that down-regulation of GPS1 expression in ESCC cells significantly inhibited the proliferation, migration and invasion of ESCC cells (Xiong et al. 2023), and gene microarray assay revealed that GPS1 expression was significantly higher in gliomas than in non-tumor tissues (Scrideli et al. 2008), and whole-genome sequencing revealed that GPS1 was mutated in penile squamous cell carcinoma (Feber et al. 2016). In breast cancer, although GPS1 has been shown to be associated with WBP2-mediated activation of the Wnt signaling pathway (Li et al. 2018), its specific molecular regulatory mechanism in breast cancer is unclear. Therefore, in this study, we performed a comprehensive bioinformatics analysis of GPS1 expression in breast cancer using the Cancer Genome Atlas (TCGA) public database, the Tumor Immunity Evaluation (TIMER) database, and the UALCAN database; and assessed the prognostic value of GPS1 using the Kaplan–Meier Plotter database. To further understand the pathogenic mechanism of GPS1 in breast cancer, we explored GPS1 co-expressed genes through GO and KEGG databases, analyzed the interaction of GPS1 protein with other proteins using STRING database (https://cn.string-db.org/), and estimated the relationship between GPS1 expression and immune cells using TISIDB database infiltration. The biological function of GPS1 in breast cancer cell lines MCF7 and MDA-MB-231 was studied by down-regulating the expression of GPS1 by siRNA in vitro. In this study, we investigated the mechanisms by which GPS1 regulates the malignant biological behavior of breast cancer, its potential prognostic value and its relationship with immune cell infiltration, to elucidate the molecular mechanism in the development of breast cancer and identify potential new targets for breast cancer therapy.

## Materials and methods

### Gene expression and prognostic analysis

The TIMER database (http://timer.cistrome.org) was used to assess the expression levels of GPS1 in 33 cancers from the TCGA dataset. The TCGA-BRCA dataset was collected using the TCGA database (https://portal.gdc.cancer.gov/), including the GPS1 gene expression matrices of 1,113 breast cancer cancer tissues and 113 normal tissues. T-tests were used to perform unpaired and paired analyses of breast cancer and paracancer tissues, and COX regression was applied to analyze the association between GPS1 and clinicopathologic characteristics of breast cancer patients. Expression of GPS1 in different subgroups of breast cancer and in the TP53 mutation state was assessed using the "TCGA gene analysis" module of the UALCAN database (http://ualcan.path.uab.edu/). The relationship between GPS1 expression and breast cancer survival was analyzed using the Kaplan–Meier Plotter database (https://kmplot.com/analysis/) and the GEPIA database (http://gepia.cancer-pku.cn/).

### Co-expressed genes and regulatory networks of GPS1

307 co-expressed genes with GPS1 Pearson correlation coefficient > 0.3 in breast cancer were selected using the UALCAN database. They were uploaded to Xiantao Academic (https://www.xiantaozi.com/) for KEGG pathway enrichment and GO functional analysis. Interaction networks of proteins were analyzed using the STRING database (https://cn.string-db.org/) and GO/KEGG enrichment was performed using Xiantao Academic.

### Immune infiltration analysis

Based on the TCGA-BRCA cohort, we evaluated the expression of GPS1 in different immunostratified breast cancers using the TISIDB database (http://cis.hku.hk/TISIDB/) and analyzed the correlation of GPS1 expression with TIIC, chemokines, chemokine receptors, and immunosuppressive molecules.

### Cell culture and siRNA transfection

We cultured human breast cancer cell lines MCF7 and MDA-MB-231 cells in a relevant complete medium containing 10% FBS, and placed them in a 37℃, 5% CO_2_ constant temperature and humidity incubator. Prior to transfection, cells were cultured in 6-well plates to approximately 50% density. Small interfering RNA (siRNA) and negative control were transfected into MDA-MB-231 cells using Lipo8000™. The transfection efficiency of the cells was detected by RT-qPCR after 24 h of transfection, and the cells were collected for the subsequent experiments. siRNA sequences were as follows: si-GPS1-F: CUGGCUUCCUUUGAUCACUTT; si-GPS1-R: AGUGAUCAAAGGAAGCCAGTT; siRNA-NC-F: 5'-UUCUCCGAACGUGUCACGUTT-3'; siRNA-NC-R: 5'- ACGUGACACGUUCGGAGAATT -3'.

### CCK-8 cell proliferation assay

Collect the cells of each group in logarithmic growth phase for the experiment, inoculate the cells in 96-well cell culture plate(100 μl/well, 3 replicate), and set up a blank group (no cells, only culture medium), placed in 37 ℃, 5% CO_2_ incubator for culture, the cells affixed to the wall were incubated from day 1 to day 5, respectively, using the culture medium 10% CCK-8 for 2 h, and detected the absorbance value at 450 nm.

### Transwell cell invasion and migration assay

Matrigel gel diluted with FBS-free medium at a ratio of 4:1 was spread evenly in 24-well Transwell chambers to assess tumor invasiveness, while the upper chamber without Matrigel coating was used to assess tumor cell migration. The upper chamber was added at 4 × 10^4^ cells/100 μl per well, and 600 μl of medium containing 10% FBS was added to the lower chambers. At the end of the culture, the invasive/migrating cells were washed with PBS, fixed with 4% paraformaldehyde for 30 min, and immersed with 1% crystal violet dye for 60 min. The number of cells in five different sites was counted by observation under the microscope.

### Statistical analysis

SPSS 27 and Prism GraphPad 7.0 software were used for statistical analysis. Two-group comparisons of continuous variables were performed by t-test. Kruskal–Wallis test and Wilcox test were used for non-parametric tests. Potential prognostic factors were screened using univariate and multivariate Cox analyses.* p* < 0.05 was considered statistically significant (* for *p* < 0.05; ** for *p* < 0.01; *** for *p* < 0.001).

## Results

### Transcript levels of GPS1

**Fig. 1:**
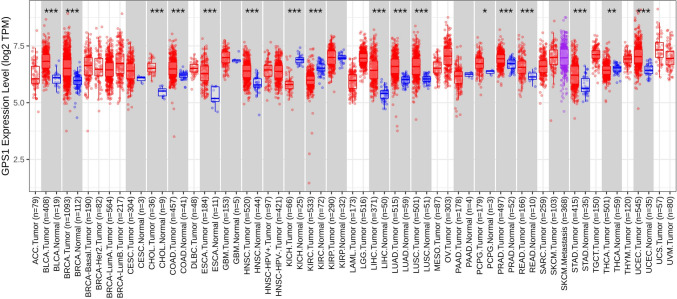
GPS1 pan-cancer analysis. **A** GPS1 expression was significantly higher in cancer tissues than in unpaired paraneoplastic tissues (*p* < 0.001). **B** GPS1 expression was significantly higher in cancer tissues than in paired paraneoplastic tissues ( *p* < 0.001)

**Fig. 2:**
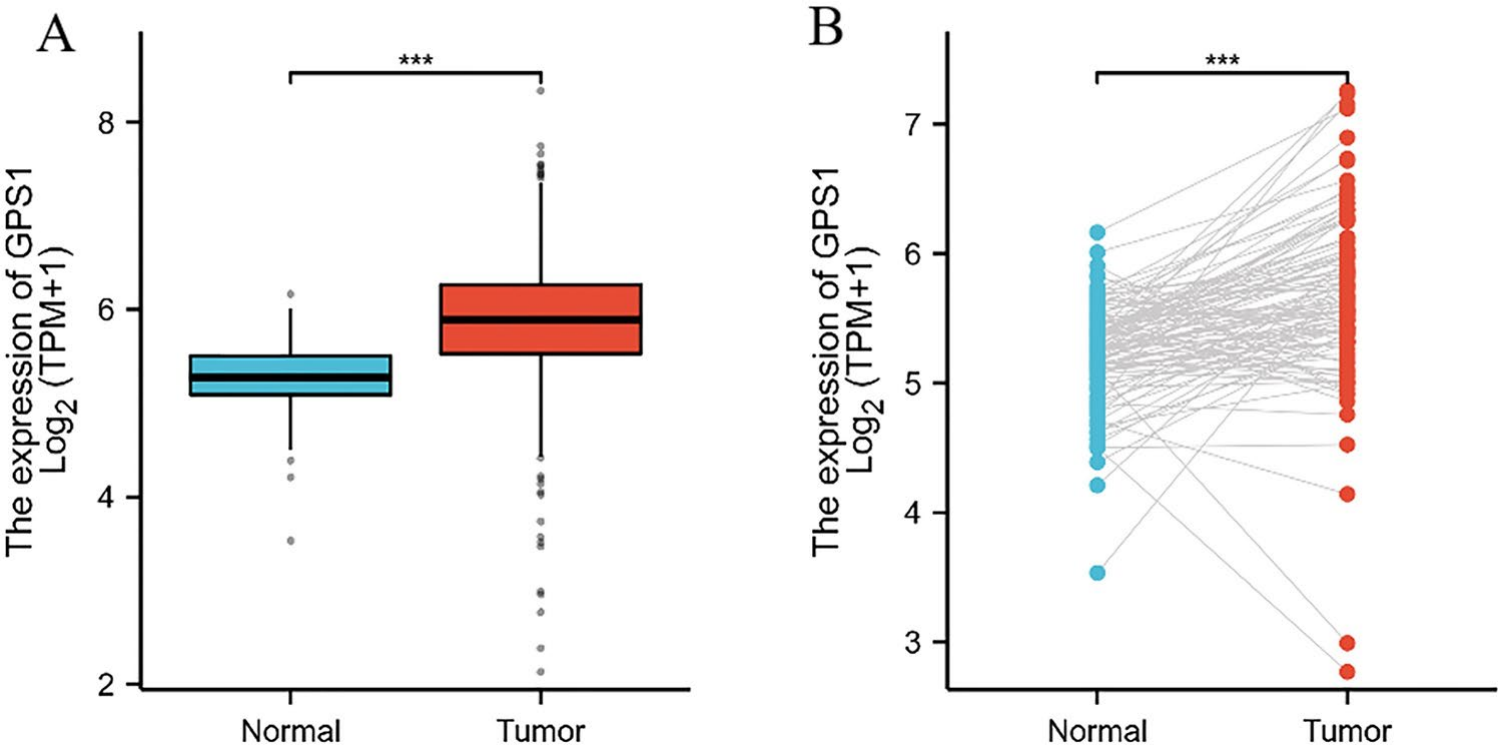
Differential expression analysis of GPS1 gene in breast cancer tissues and corresponding paracancerous tissues based on the TCGA database. **A** The expression of GPS1 in major subtypes of breast cancer was significantly higher than that in normal tissues (*p* < 0.05). **B** The expression level of GPS1 in samples with TP53 mutation was higher than that in samples without mutation (*p* < 0.05)

**Fig. 3:**
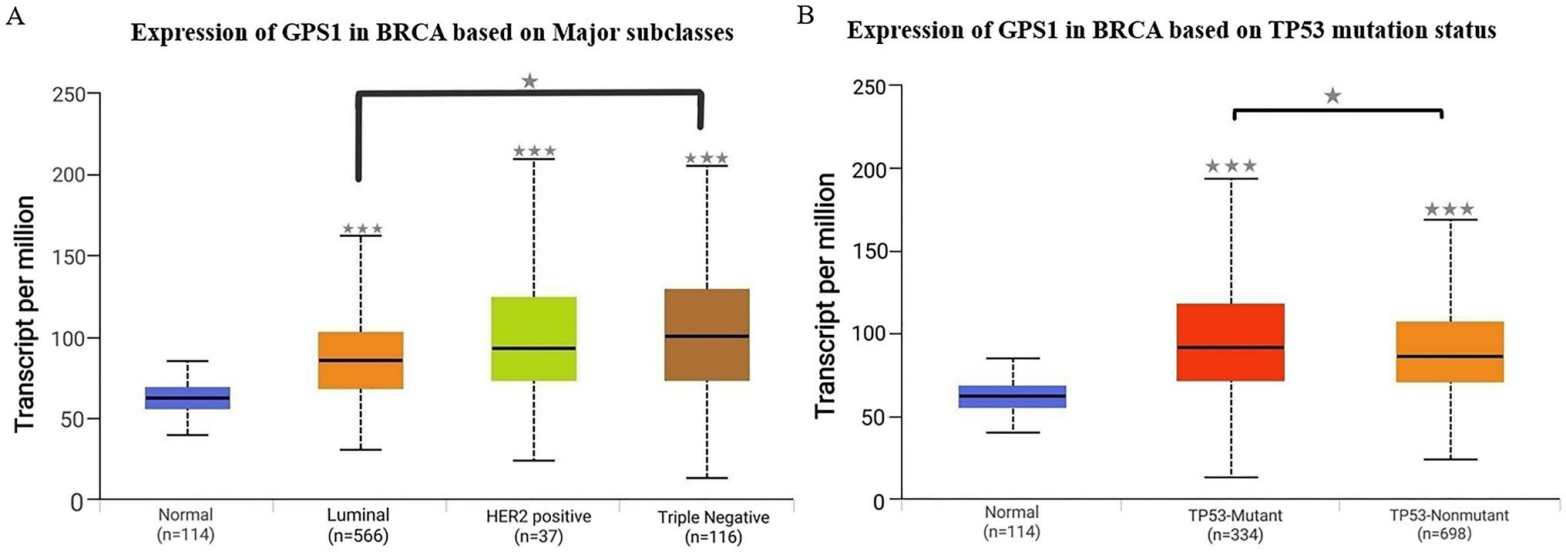
Subgroup analysis of the UALCAN database and expression analysis in the TP53 mutation state

The expression status of GPS1 in pan-cancer was estimated using the TIMER database (Fig. 1). The results showed that GPS1 was highly expressed in most cancers, such as BLCA, BRCA, CHOL, ESCA, LIHC, and was lowly expressed in a few cancers, such as KICH, KIRC, and THCA. Differential expression of GPS1 in breast cancer was further analyzed using the TCGA-BRCA dataset, which showed that GPS1 was highly expressed in breast cancer tissues (5.88 ± 0.64) compared with normal tissues (5.24 ± 0.39) in the unpaired analysis (*p* < 0.001, Fig. 2A), and in breast cancer tissues (5.67 ± 0.68) compared with paracancerous tissues (5.24 ± 0.39) in the paired analysis (*p* < 0.001, Fig. 2B). Subgroup analysis using the UALCAN database showed that the expression of GPS1 was significantly higher than that in normal tissues in all subtypes of breast cancer, including luminal, Her2-positive and triple-negative breast cancers, with the expression in triple-negative breast cancers significantly higher than that in luminal types (*p* < 0.05, Fig. 3A). This result suggests that GPS1 may play different roles in different subtypes of breast cancer. TP53 mutations are present in 30% of breast cancer patients, and the complex interactions of the TP53 signaling pathway with other signaling pathways can lead to differential effects on breast cancer cell behavior. To understand whether GPS1 plays a role in regulating TP53 activity, we analyzed the correlation between TP53 mutation status and GPS1 expression, and our results showed that GPS1 levels were higher in TP53-mutant breast cancers compared to TP53-free breast cancers (P < 0.05, Fig. 3B).

### Correlation between GPS1 expression and clinical features

**Fig. 4:**
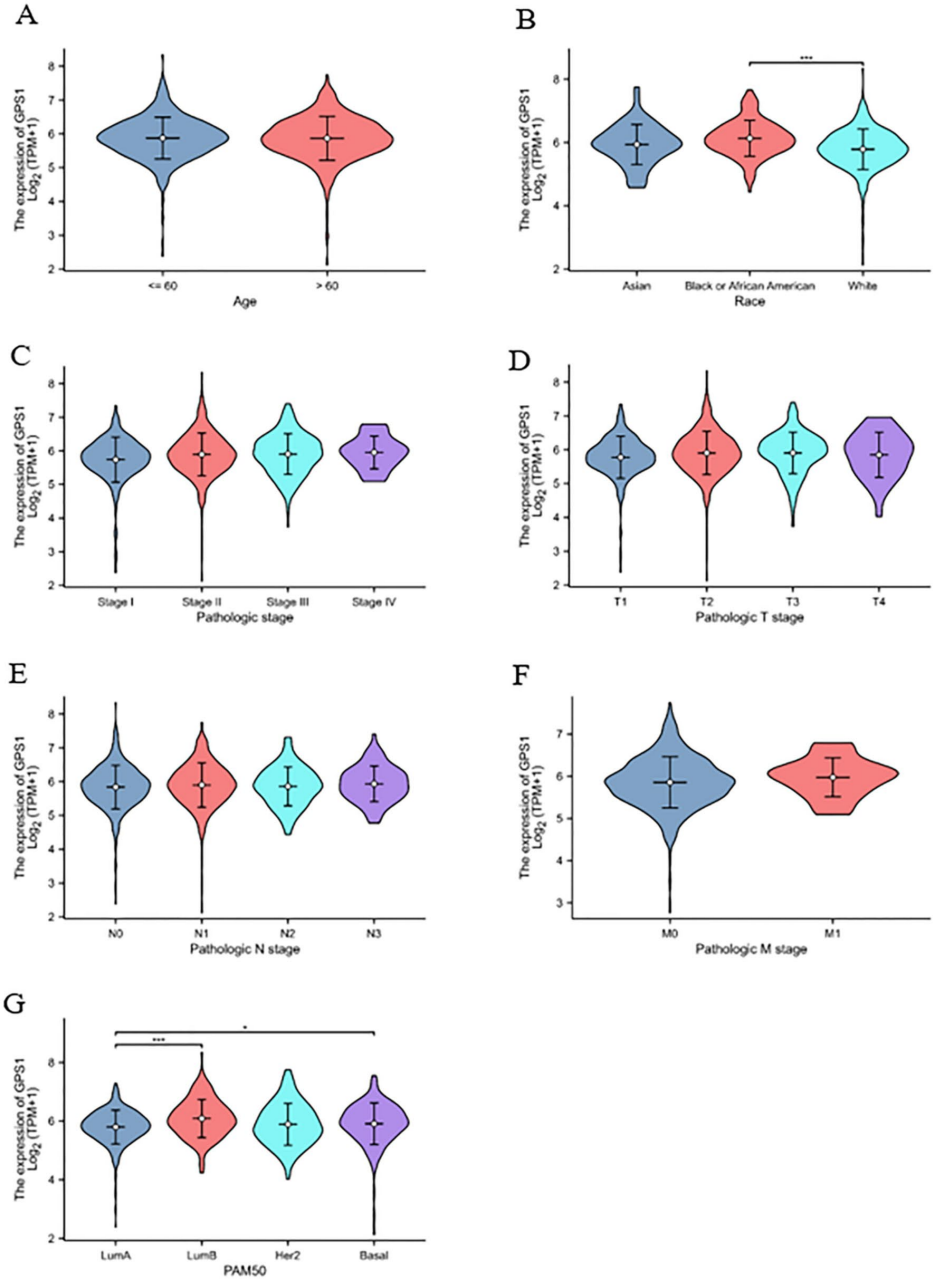
Correlation of GPS1 expression with clinicopathologic parameters. **A** Age (Wilcox test). **B** Race (Kruskal–Wallis test). **C** Clinical staging (Kruskal–Wallis test). **D** T staging (Kruskal–Wallis test). **E** N staging (Kruskal–Wallis test). **F** M staging (Wilcox test). **G** PAM50 staging (Kruskal–Wallis test)

**Fig. 5:**
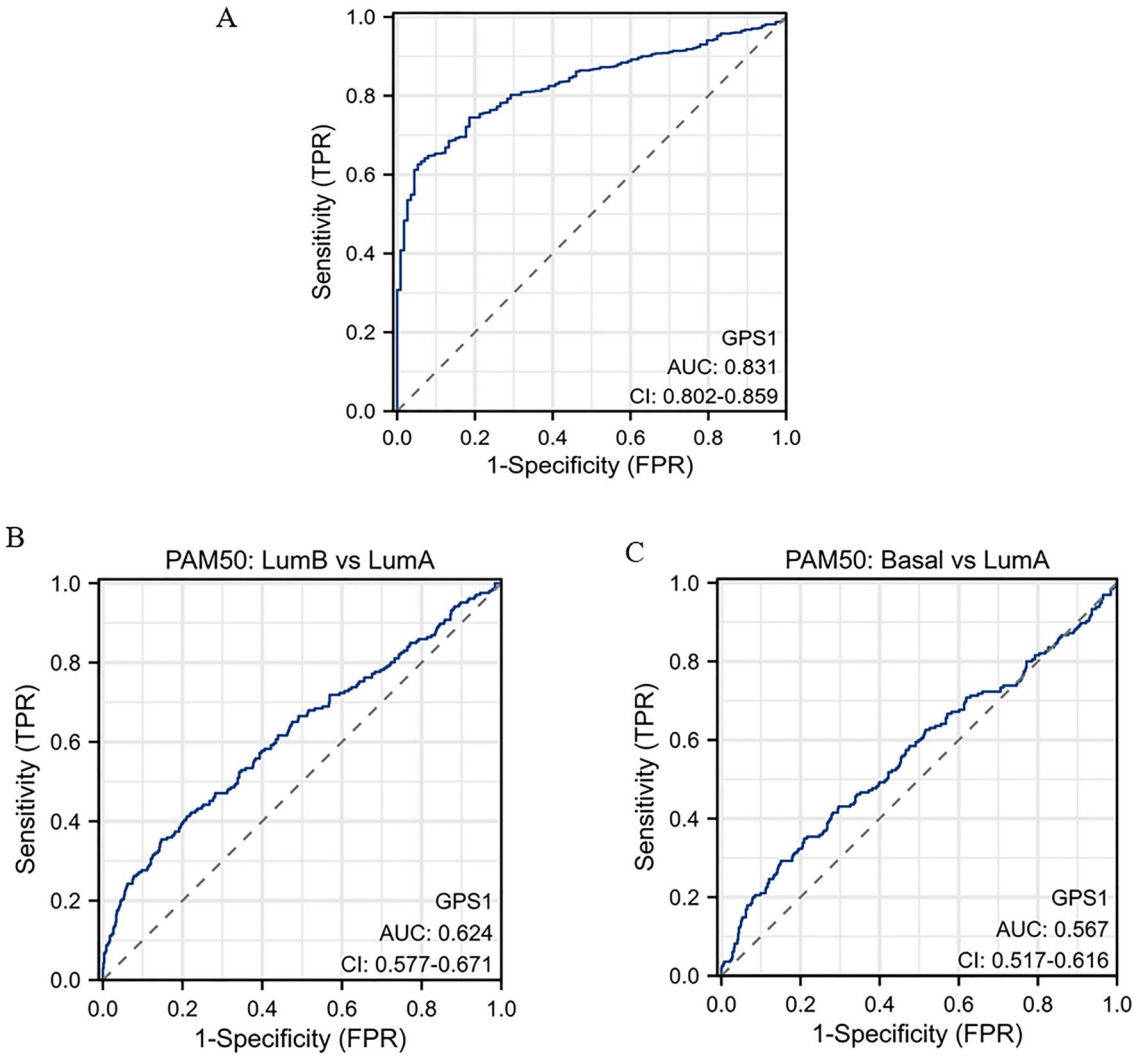
Diagnostic ROC curves for GPS1 prediction of breast cancer and subtypes. **A** GPS1 predicts diagnostic ROC curves for breast cancer. **B** GPS1 predicts diagnostic ROC curves for Luminal A and Luminal B breast cancers in PAM50 subtypes. **C** GPS1 predicts diagnostic ROC curves for Luminal A and Basal breast cancers in PAM50 subtypes

Clinical correlation analysis showed that elevated GPS1 expression was significantly correlated with race (Fig. 4B, p < 0.001) and PAM50 staging (Fig. 4G, p < 0.001), whereas the correlation with the parameters of age, clinical and pathological staging was not significant (Fig. 4A, C, D, E, F). GPS1 expression was significantly higher in black and African American than in Caucasian (Fig. 4B, p < 0.001), which may be related to differences in gene expression between races. PAM50 typing is a molecular typing method for subtyping breast cancer based on its mRNA expression matrix, which allows for more accurate subtyping of highly heterogeneous breast cancers, thereby effectively predicting the unique molecular features, prognosis, clinical behavior, and therapeutic response of each subtype. The results of this study showed that the expression of GPS1 in Luminal B and Basal type breast cancer was significantly higher than that in Luminal A type (*p* < 0.05, Fig. 4G). Further, we analyzed the predictive value of GPS1 as a diagnostic indicator in the diagnosis of breast cancer and its staging. Data were downloaded from the TCGA database (https://portal.gdc.cancer.gov) and RNAseq data from the TCGA-BRCA (Breast Cancer) project were organized. Data in TAM format was extracted and ROC analysis was performed on the data using the pROC package, and the results were visualized using ggplot2. The receiver operating curve (ROC) revealed that GPS1 had some accuracy in predicting breast cancer (AUC = 0.832, Fig. 5A) and could be used as an adjunctive diagnosis of breast cancer, but was less accurate in determining breast cancer staging (AUC < 0.7, Fig. 5B–C).

### Increased GPS1 expression relates to unfavorable prognosis in BC patients

**Fig. 6:**
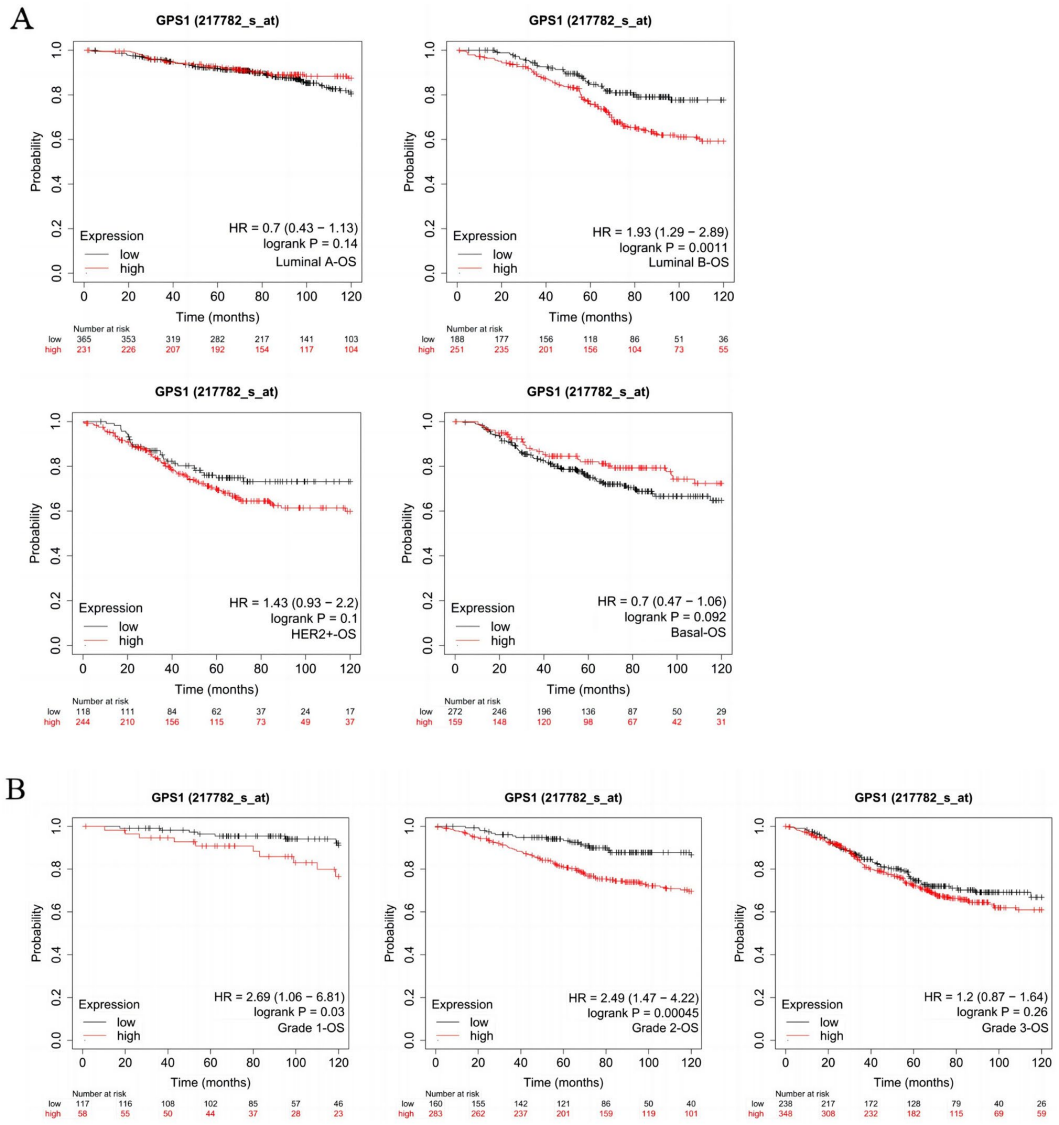

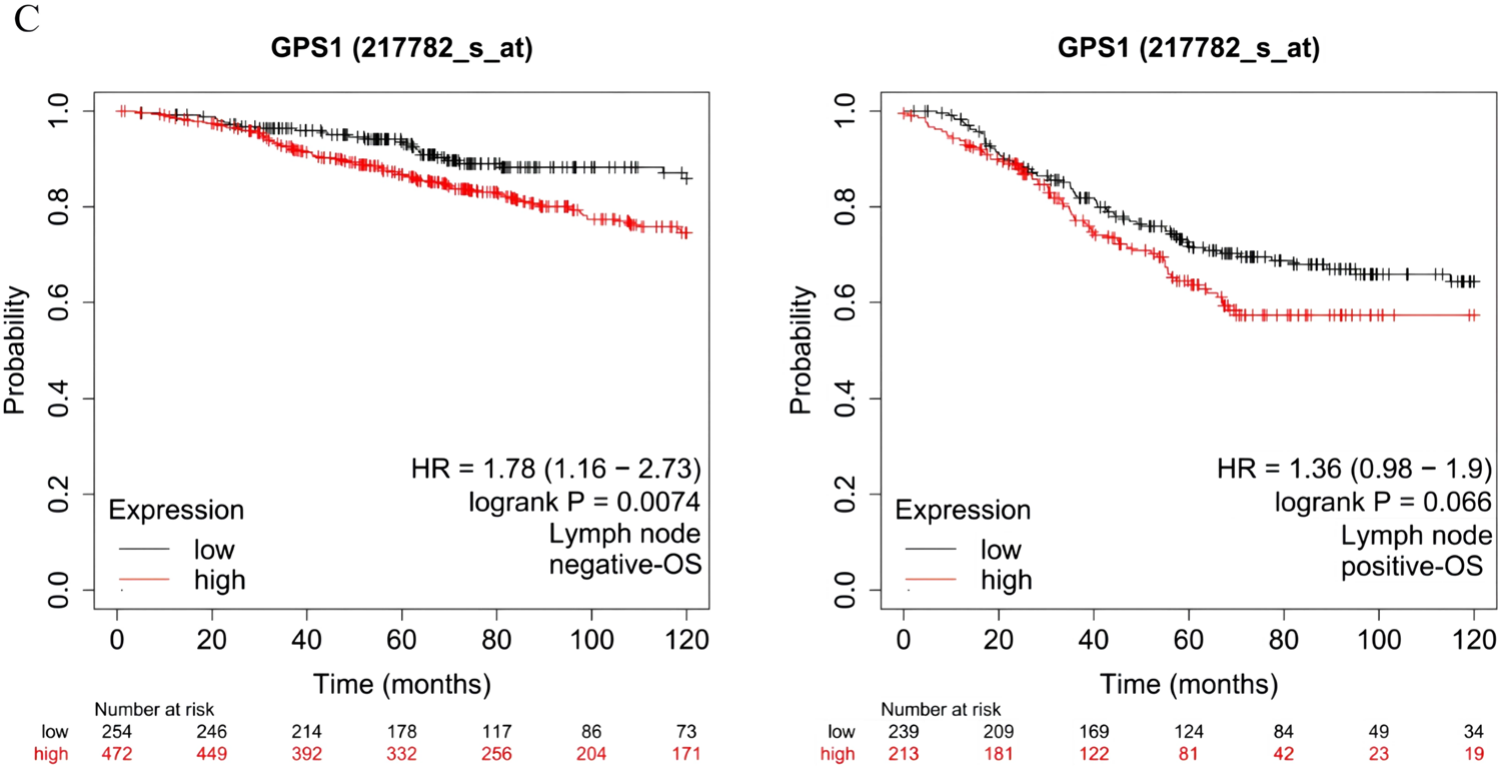
Correlation between GPS1 expression and prognosis. **A** The effect of GPS1 on breast cancer prognosis in different breast cancer subtypes. **B** The effect of GPS1 on breast cancer prognosis in different clinical grades. **C** The effect of GPS1 on breast cancer prognosis in different lymph node metastasis status

**Fig. 7:**
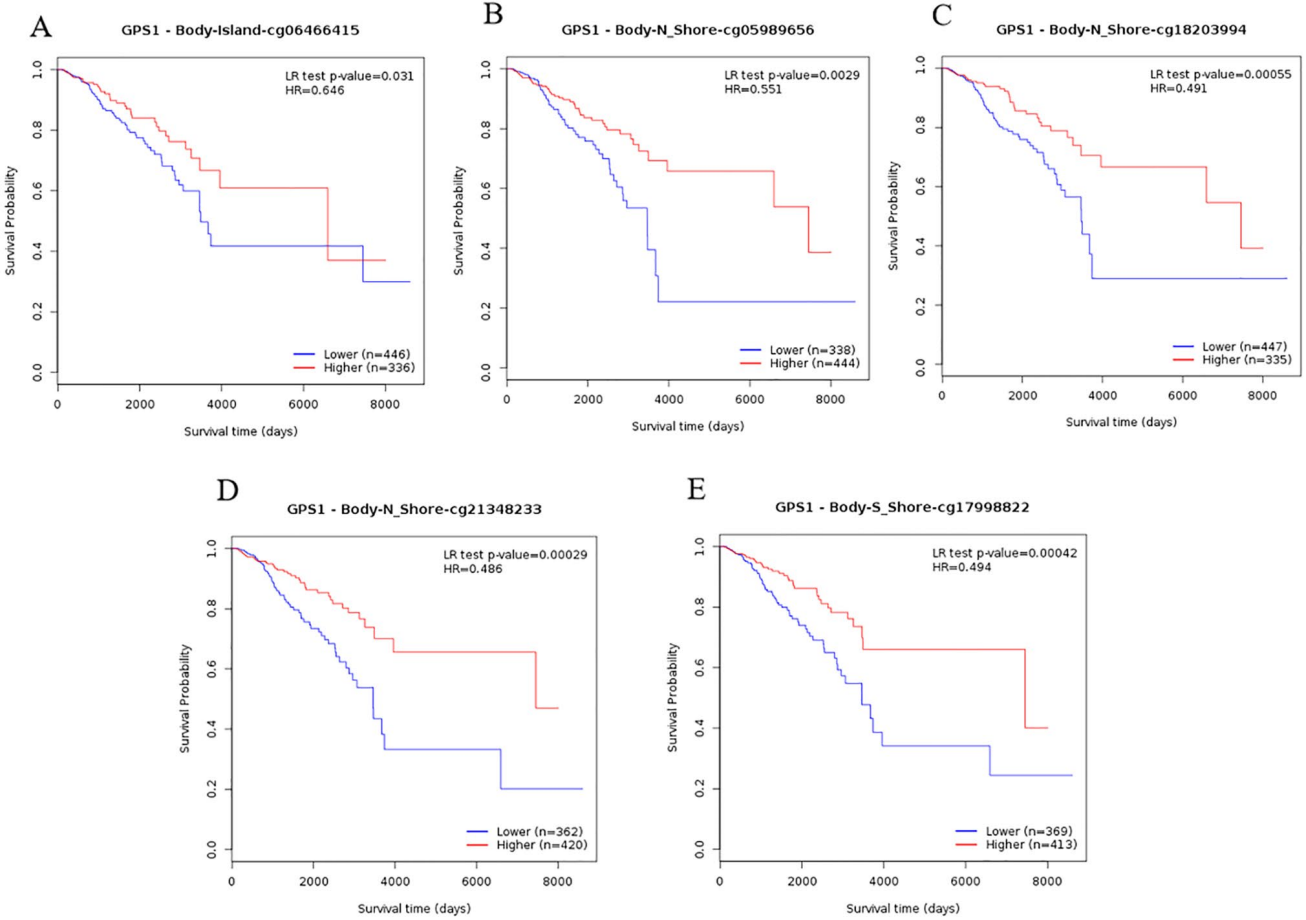
Correlation between methylation site status and prognosis of GPS1. **A** Hypomethylation at cg06466415 suggests a poor prognosis. **B** Hypomethylation at cg05989656 suggests a poor prognosis. **C** Hypomethylation at cg18203994 suggests a poor prognosis. **D** Hypomethylation at cg21348233 suggests a poor prognosis. **E** Hypomethylation at cg17998822 suggests a poor prognosis

Kaplan–Meier Plotter database was used to evaluate the impact of GPS1 on the prognosis of breast cancer in terms of the four main subtypes of breast cancer, the presence or absence of lymph node metastasis, and pathologic grading. The results showed that the prognostic value of GPS1 was significant in Luminal B breast cancer, and the prognosis was worse in those with high expression of GPS1 (*p* < 0.01), whereas no significant prognostic significance was found in Luminal A, Basal-like, and HER2 + breast cancers (Fig. 6A). High expression of GPS1 in different pathologic grades of breast cancer suggests poor prognosis, and this difference is particularly significant in low- and intermediate-grade breast cancers (Fig. 6B). High expression of GPS1 in patients was significantly negatively correlated with prognosis (Fig. 6C), and the difference was significant only in the group without lymph node metastasis. Further, we also analyzed the relationship between methylation and prognosis of breast cancer, we analyzed the effect of GPS1 methylation status on the prognosis of breast cancer using the GEPIA database. Subgroup analysis based on the median methylation degree revealed that the overall survival rate of hypomethylated patients was lower than that of hypermethylated patients (*p* < 0.05), and the hypomethylation of five CpG loci, cg06466415, cg05989656, cg18203994, cg21348233, and cg17998822, suggested a poor prognosis (Fig. 7 A-E).

### Gene enrichment analysis

**Fig. 8:**
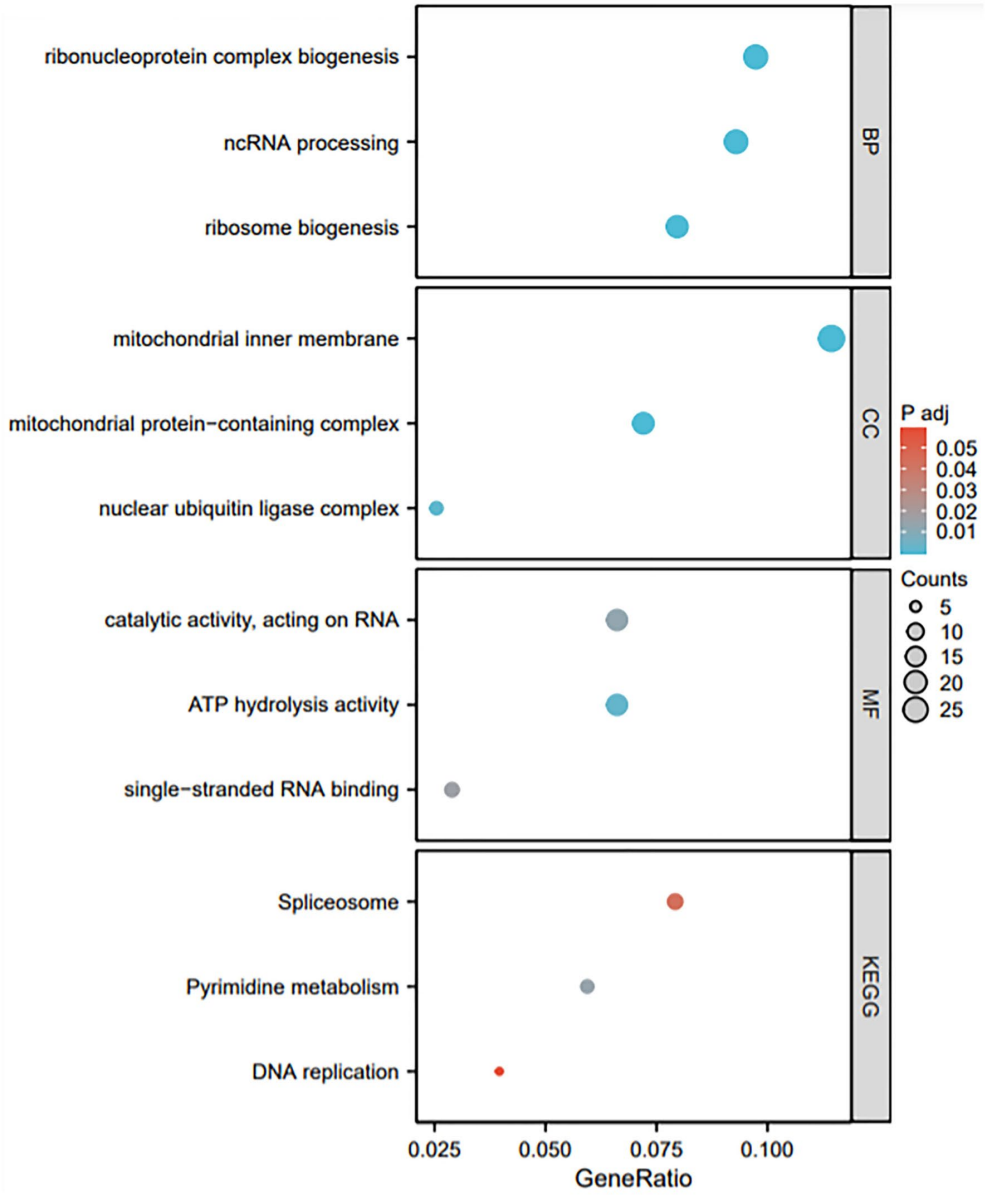
GO-KEGG enrichment analysis of GPS1

In general, co-expressed genes have similar functions and roles. We utilized the UALCAN database to screen the GPS1 co-expressed genes in the TCGA-BRCA cohort for GO and KEGG pathway analyses (Fig. 8). GO analysis showed that the key biological processes (BP) were those involved in ribonucleoprotein complex biogenesis, non-coding RNA processing and ribosome biogenesis. The cellular components (CC) were components of the inner mitochondrial membrane, mitochondrial protein complex and nuclear ubiquitin ligase complex. The genes coexpressed with GPS1 mainly have molecular functions (MF) such as catalyzing RNA, hydrolyzing ATP and binding single-stranded RNA. As for KEGG pathways, the results suggest that GPS1 co-expressed genes are mainly associated with splicer formation, pyrimidine metabolism and DNA replication, which are critical processes in BC development.

### Protein interaction and enrichment analysis

**Fig. 9:**
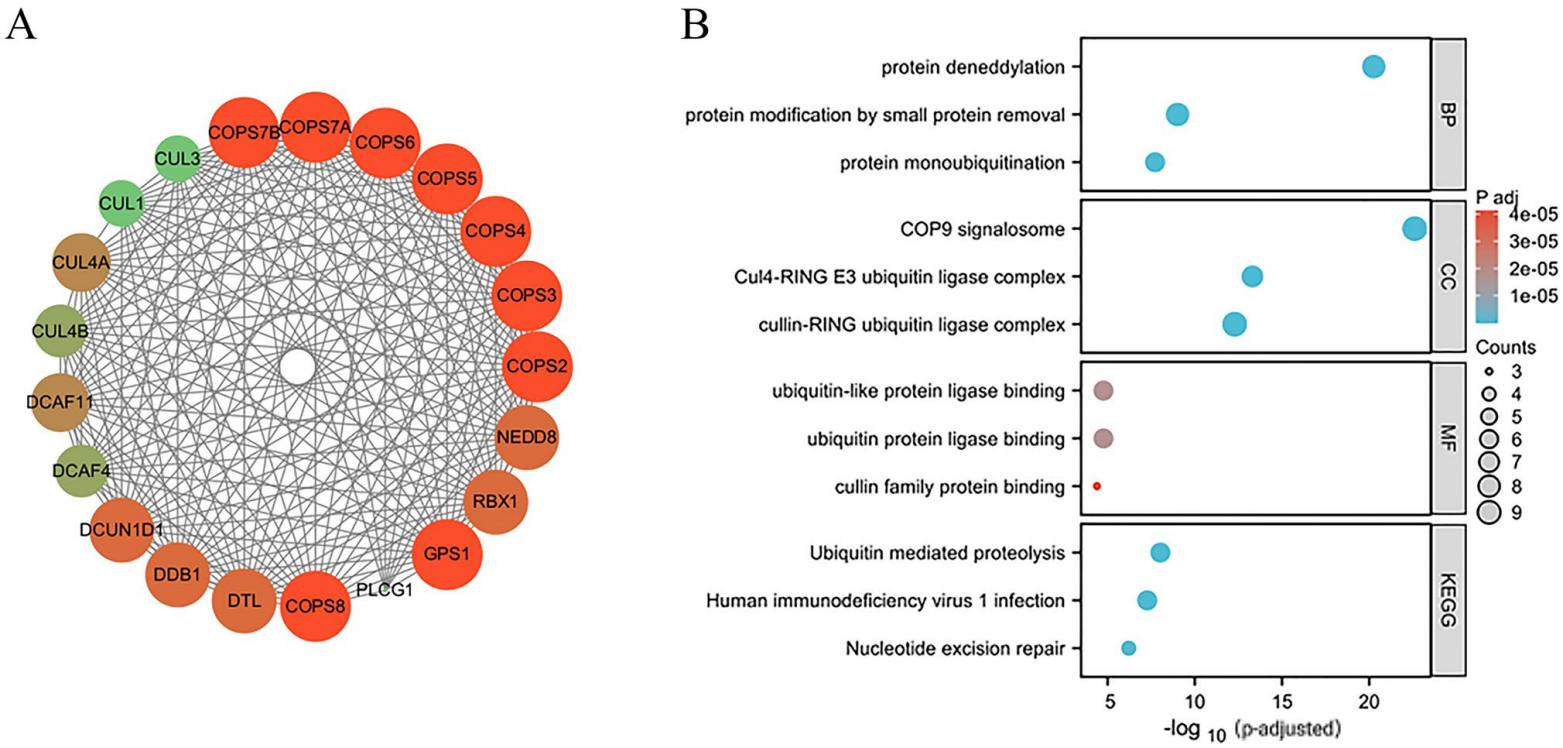
Potential network of GPS1 interaction with other proteins and GO/KEGG enrichment analysis. **A** Potential network of GPS1 interaction with other proteins. **B** GO-KEGG enrichment analysis of GPS1 interaction with other proteins

The interaction of the GPS1 protein with other proteins was analyzed by the STRING database (https://cn.string-db.org/). The network relationship diagram showed that GPS1 may interact with 20 proteins including COPS2-8, DTL, DCAF4, DCAF11, etc. (Fig. 9A). To further understand the function of GPS1 protein, GO-KEGG enrichment analysis was performed (Fig. 9B). GO analysis showed that the key biological processes (BPs) were cenoprotein deformylation, mono-ubiquitination, and so on. The key cellular components (CC) were COP9 signalosome, Cul4-RING E3 ubiquitin ligase complex and cullin-RING ubiquitin ligase complex. The genes co-expressed with GPS1 mainly have molecular functions (MF) of binding to ubiquitin-like protein ligases, ubiquitin-protein ligases, and cullin-family proteins. KEGG analysis showed that GPS1 is associated with ubiquitin-mediated protein hydrolysis, nucleotide excision repair, and human immunodeficiency virus type Ι infection, among other pathways.

### Relationship between GPS1 expression and immune microenvironment

**Fig. 10:**
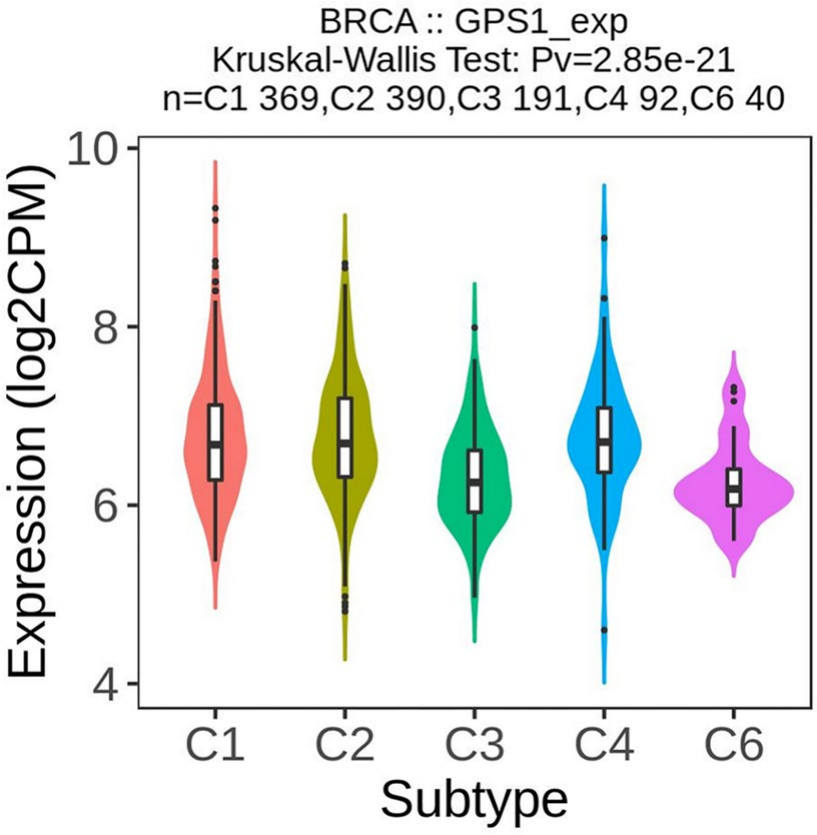
Correlation of GPS1 expression with immunologic subtypes of breast cancer

Tumor microenvironment (TME) is a heterogeneous and complex organization composed of tumor cells, stroma and endothelial cells, in which immune infiltrating cells, chemokines, chemokine receptors and immune factors constitute the immune microenvironment. The tumor immune cluster is divided into six immune subtypes, namely C1: wound healing, C2: IFN-γ dominant, C3: inflammatory, C4: lymphocyte depleted, C5: immunologically quiet and C6: TGF-β dominant. In the TISIDB database, GPS1 was significantly associated with immunological subtypes in breast cancer patients (*p* < 0.001, Fig. 10).

**Fig. 11:**
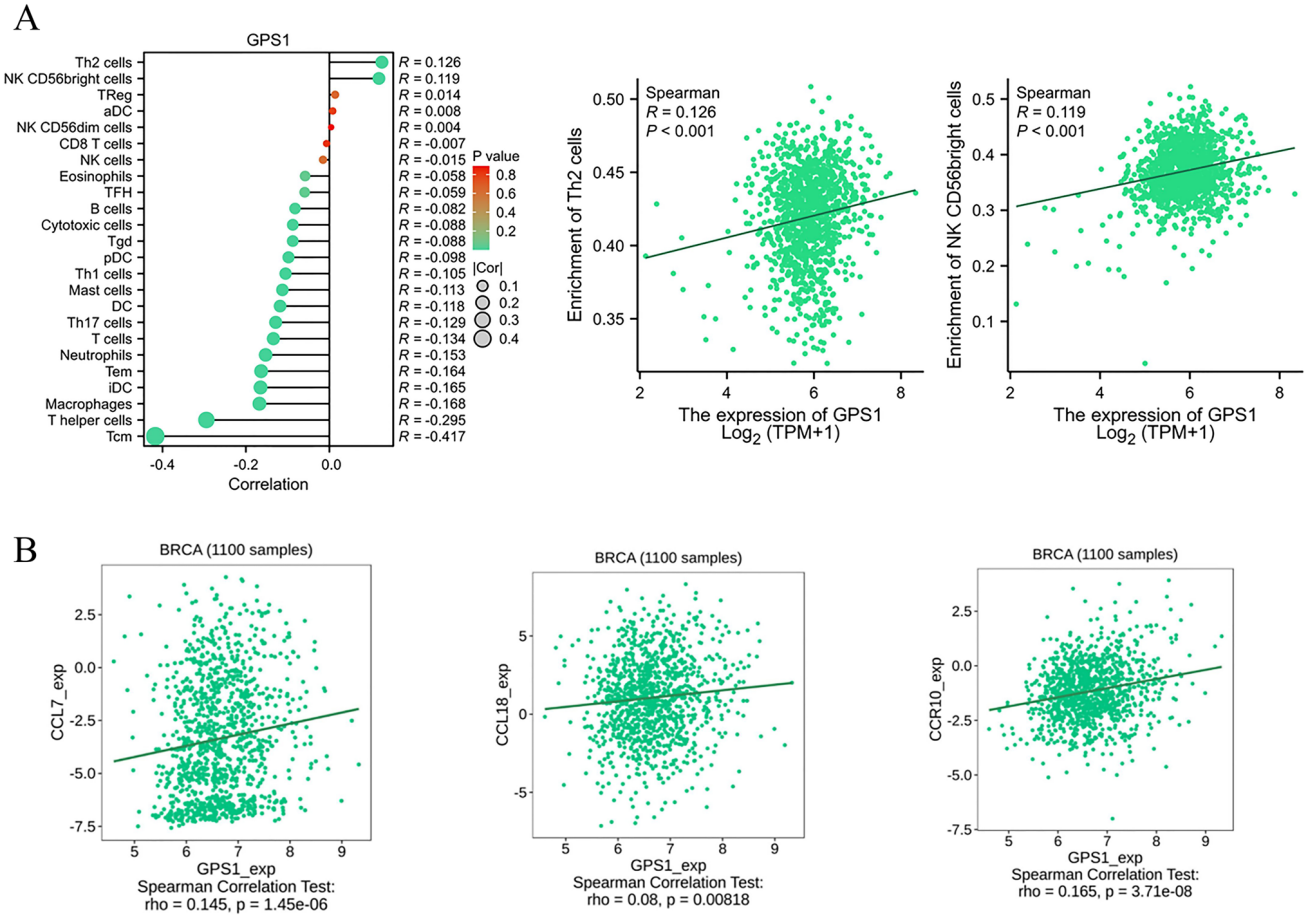
Correlation analysis of GPS1 expression with expression of immune cells, chemokines and their receptors. **A** Correlation analysis of GPS1 expression with TIICs. **B** Correlation analysis of GSDME expression with chemokine and its receptor expression

Immune cells in the immune microenvironment play an important role in suppressing or promoting tumor growth, and chemokines and chemokine receptors play a very critical role in the immune regulation process, which can recruit immune cells into the TME and influence tumor progression. TISIDB database was used to analyze the relationship between GPS1 expression and TIICs, chemokines and chemokine receptors. The results showed that GPS1 expression was positively correlated with Th2, NK, and CD56bright and negatively correlated with various immune cells such as Tcm, T helper, Macrophages, iDC, etc. (Fig. 11A). Besides, GPS1 was positively correlated with expression of chemokines CCL7 and CCL18 and chemokine receptor CCR10 (Fig. 11B). These results suggest that GPS1 influences the development of breast cancer by regulating immune cells and could be a new immunotherapy target.

**Fig. 12:**
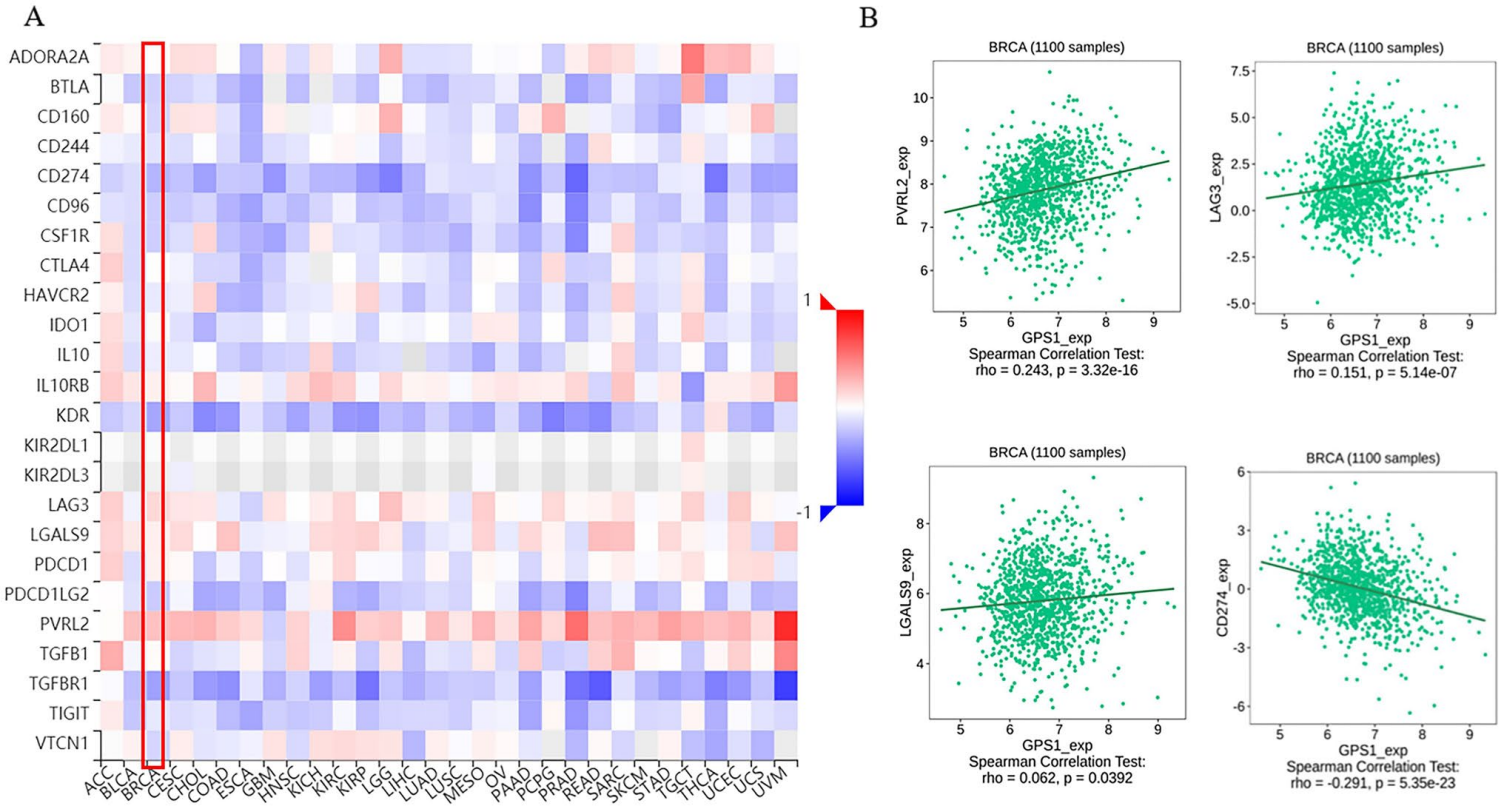
Correlation analysis of GPS1 expression with immunosuppressive sites. **A** Heatmap of the relationship between GPS1 expression and inhibitory immune checkpoints. **B** Positive correlation between GPS1 expression and LAG3, PVRL2, LGALS9 expression and negative correlation with CD274 expression

In the tumor microenvironment (TME), increased expression of inhibitory immune checkpoints and elevated immunosuppressive cytokines correlate with the formation and maintenance of immunosuppressive microenvironment and promote the escape of tumoral cells. Therefore, in this paper, we assessed the correlation between GPS1 expression and immune inhibitors in breast cancer through the TISIDB database. The heatmap showed the relationship between GPS1 expression and inhibitory immune checkpoints in tumors (Fig. 12A). The results showed that in breast cancer, GPS1 expression was positively correlated with LAG3, PVRL2, and LGALS9 (Fig. 12B), and negatively correlated with CD274 and HAVCR2, but not with CTLA4 and PDCD1. These results suggest that GPS1 regulates immunosuppressive sites to inhibit the immune response in breast cancer, thus affecting immune cell infiltration.

### Inhibition of GPS1 expression inhibits cell proliferation, invasion and migration

**Fig. 13:**
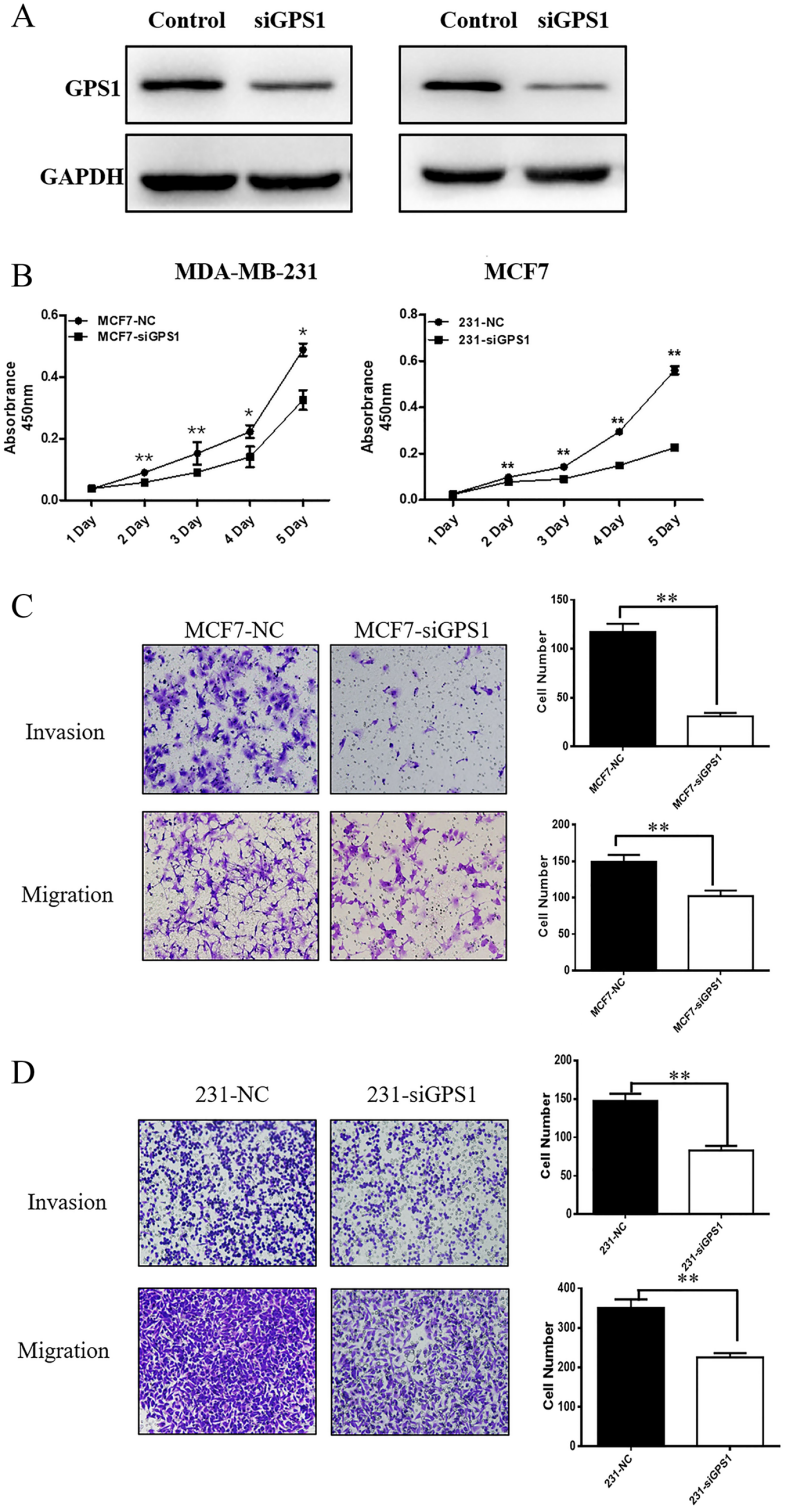
In vitro knockdown of GPS1 inhibits proliferation, invasion and migration of breast cancer cells. **A** GPS1 protein expression level was significantly reduced in siRNA knockdown cell lines. **B** Cell proliferation viability was monitored by CCK-8 assay, and tumor cell viability was significantly suppressed in the GPS1 knockdown group. **C** Transwell invasion and migration assay showed that invasion ability was significantly reduced in the GPS1 knockdown group in the MCF7 cell line. **D** Transwell invasion and migration assay showed that the MDA-MB-231 cell line showed significantly reduced invasion ability in the GPS1 knockdown group

To elucidate the role of GPS1 in breast cancer progression, we investigated the biological function of GPS1 in MCF7 and MDA-MB-231 cells by knocking down the expression of GPS1 by siRNA. RT-qPCR results showed that, compared with the Control group, siRNA significantly reduced the GPS1 expression in MCF7 and MDA-MB-231 cells levels (Fig. 13A). The results of cell proliferation assay showed that GPS1 knockdown significantly inhibited the cell viability of MCF7 and MDA-MB-231 compared with Control group (Fig. 13B). In addition, we evaluated the effects of GPS1 on breast cancer cell migration and invasion. Transwell invasion and migration assays showed that GPS1 knockdown significantly inhibited the number of invasive and migrating cells in MCF7 and MDA-MB-231 cells compared with the NC group (Fig. 13C and D). These data suggest a pro-carcinogenic role of GPS1 in breast cancer.

## Discussion

Currently, only a few studies have reported the function of GPS1 in cancer (Xiong et al. 2023, Li et al. 2018, Feber et al. 2016, Scrideli et al. 2008). In this study, we performed a comprehensive bioinformatics analysis to investigate the expression profile, prognostic value, biological function, and potential regulatory pathways of GPS1 in breast cancer. Based on the bioinformatics analysis, we found that GPS1 expression was upregulated in breast cancer and was associated with race, PAM50 typing, TP53 mutation and methylation status. We found higher levels of GPS1 in TP53-mutant breast cancers than in breast cancers without TP53 mutations. This finding was validated in our subsequent protein immunoblotting assay of multiple cell lines, where the expression of GPS1 protein was significantly higher in the T47D cell line harboring mutated nonfunctional TP53 than in the MCF-7 cell line without TP53 mutation. It has been reported that CSN-specific phosphorylation of TP53 dedicates the protein to rapid degradation by the ubiquitin-26S proteasome system (Bech-Otschir et al. 2001), and thus we hypothesized that GPS1 could contribute to breast cancer development by affecting the stability and mutational status of TP53. Further receiver operating curve (ROC) analysis showed that GPS1 had a certain degree of accuracy in predicting the occurrence of breast cancer; prognostic analysis revealed that those with high expression of GPS1 had a worse prognosis in Luminal B, low to intermediate-grade breast cancers; and there was a significant negative correlation between the expression of GPS1 and the prognosis of patients without lymph node metastasis. These results suggest that GPS1 may play an important role in the early stage of breast cancer development and is expected to be a biomarker for early diagnosis of breast cancer. In addition, the analysis of the effect of GPS1 methylation status on the prognosis of breast cancer revealed that the hypomethylation status of the five methylation sites may be an important factor affecting the prognosis, and it can be used as an effective biomarker to identify the poor prognosis of breast cancer.

To further investigate the biological function of GPS1 in breast cancer, we characterized the co-expressed genes and related pathways of GPS1. The results showed that GPS1 was positively correlated with the highest co-expressed genes, DUS1L, STRA13, CCDC137, MRPL12, and ANAPC11. Among them, MRPL12, as a component of the mitochondrial ribosomal protein complex (Hu et al. 2023), is an RNA-binding protein, and in vitro experiments demonstrated that down-regulation of its endogenous expression significantly inhibited the proliferation and migration of breast cancer (Liu et al. 2021a, b); its expression pattern was correlated with the poor prognosis of the patients. Analyzing the GO and KEGG pathways, GPS1 was mainly involved in mitochondrial ribosomal biogenesis and is related to pathways such as spliceosome formation and pyrimidine metabolism. Accordingly, we speculate that GPS1 may be a transcriptional co-activator at the gene level, which participates in the formation of ribonucleoprotein complexes by binding to the modular or structural motifs of target RNAs, thereby dynamically altering the fate or function of RNAs, such as regulating their stability, localization, modification and translation.

Notably, WBP2, a gene co-expressed at position 68 of GPS1, is an emerging oncogene that has received recent attention for its oncogenic role in TNBC breast cancers (Li et al. 2018; Lim et al. 2016). WBP2 is a node between the signaling protein Wnt and other signaling molecules and pathways including the epidermal growth factor receptor, estrogen receptor/progesterone receptor (Buffa et al. 2013; Lim et al. 2011; Chen et al. 2007; Dhananjayan et al. 2006). Studies have shown that in the breast cancer cell line MDA-MB-231, WBP2 initiates cellular Wnt activity through the upregulation of GPS1 and TNIK (Lim et al. 2016). GPS1 activates the JNK/Jun pathway (Li et al. 2007), which forms a positive feedback loop with TNIK to mediate Wnt-induced AXIN2 expression (Li et al. 2018). This finding suggests that GPS1 interacts with WBP2 to transcriptionally regulate a group of Wnt-independent target genes, which prepares the "molecular soil" for acute cellular response to Wnt and promotes the growth of breast cancer cells.

The above results demonstrated that GPS1 regulates a complex gene network, which prompted us to investigate the "molecular soil" at another level, i.e., proteomics. PPI protein interactions showed that GPS1 is mainly involved in biological processes such as protein denaturation and monoubiquitination, and is related to the pathways of ubiquitin-mediated protein hydrolysis, nucleotide excision and repair, etc. GPS1, as part of the COP9 signalosome (CSN), interacts with ubiquitin E3-ligase (RBX1) (Qin et al. 2020), which mediates ubiquitination and proteasomal degradation of target proteins that are involved in cell-cycle progression, signaling, transcription, and nucleotide excision repair of transcriptional coupling. Analysis of the proteomics data further confirmed that GPS1 can be involved in the development of breast cancer through multiple pathophysiological processes.

Next, we explored the effect of GPS1 on the immune microenvironment, and data analysis revealed that GPS1 was differentially expressed in various immune subtypes of breast cancer and was positively correlated with the infiltration of Th2, NK, and CD56bright immune cells.Th2 cells are an important immune component of the tumor microenvironment and play a central role in the type-II immune response (Lorvik et al. 2016); some studies have shown that the immunosuppressive nature of the breast cancer tumor microenvironment is positively correlated with the proportion of Th2 cell infiltration; Th2-mediated immunity suppresses CD8 + T cell infiltration and cytotoxic activity, as well as reshaping the immune landscape and ultimately suppressing immune checkpoint blockade (ICB) responses (Chen et al. 2022). Therefore, we hypothesized that GPS1 could regulate tumor immune cell infiltration to influence breast cancer development. Further exploration of the mechanism by which GPS1 expression promotes Th2 infiltration will provide a theoretical basis for improving the efficacy of immune checkpoint blockade therapy.

Not coincidentally, studies on the correlation between GPS1 and inhibitory immune checkpoints have shown that the upregulation of GPS1 expression is positively correlated with key genes such as LAG3, PVRL2, and LGALS9. The high expression of these genes in the tumor microenvironment was associated with an attenuated T cell-mediated anti-tumor immune response. Among them, LAG3 is thought to be spatially related to T cell receptor (TCR), especially CD3-TCR, and directly inhibits T cell activation through similarity(Andrews et al. 2017). LAG3 is upregulated in breast cancer tissues, especially enriched in HER2-positive patients and those with high tumor grade, indicating that high expression of LAG3 is indicative of highly malignant breast cancer (Shi et al. 2022). In addition, LAG3 is a negative regulator of T-cell activation and can inhibit the immune response of T-cells (Liu et al. 2021). As an immunotherapeutic target currently under investigation, the correlation between LAG3 and GPS1 may provide new ideas for effective immunotherapy of breast cancer.

Finally, in vitro experimental studies revealed that knockdown of GPS1 significantly inhibited the proliferation, migration and invasion ability of breast cancer cells. Based on the bioinformatics analysis and in vitro experimental studies, we concluded that GPS1 may be a potential diagnostic biomarker for breast cancer, which is closely related to immune cell infiltration, cell proliferation, migration and invasion of breast cancer. These findings need to be confirmed in clinical studies and in vivo experiments.

## Conclusions

In summary, we believe that GPS1, as a transcriptional co-activator, can promote the malignant behavior of breast cancer by interacting with WBP2 to prepare the "molecular soil" for cellular response to Wnt signaling; the interaction between GPS1 expression and the immune microenvironment may provide a new target for immunotherapy. The bioinformatics analysis and basic research in this paper will help to better understand the role of GPS1 in breast cancer and provide a reference for future research.

## Data Availability

Public datasets were obtained from TIMER (http://timer.cistrome.org), TCGA (https://portal.gdc.cancer.gov/), UALCAN database (http://ualcan.path.uab.edu/), Kaplan–Meier Plotter (https://kmplot.com/analysis/), GEPIA (http://gepia.cancer-pku.cn/), Xiantao Academic (https://www.xiantaozi.com/), STRING (https://cn.string-db.org/) and TISIDB database (http://cis.hku.hk/TISIDB/).
